# Temperature-Dependent Amplified Spontaneous Emission in CsPbBr_3_ Thin Films Deposited by Single-Step RF-Magnetron Sputtering

**DOI:** 10.3390/nano13020306

**Published:** 2023-01-11

**Authors:** Giovanni Morello, Stefania Milanese, Maria Luisa De Giorgi, Nicola Calisi, Stefano Caporali, Francesco Biccari, Naomi Falsini, Anna Vinattieri, Marco Anni

**Affiliations:** 1 CNR-IMM, Institute for Microelectronic and Microsystems Unit of Lecce, Via per Monteroni, 73100 Lecce, Italy; 2 Center for Biomolecular Nanotechnologies @UNILE, Istituto Italiano di Tecnologia, Via Barsanti, I-73010 Arnesano (LE), Italy; 3 Dipartimento di Matematica e Fisica “Ennio De Giorgi”, Università del Salento, Via per Arnesano, 73100 Lecce, Italy; 4 Department of Industrial Engineering, University of Florence, Via di S. Marta 3, 50139 Firenze, Italy; 5 Research Unit of Firenze, National Interuniversity Consortium of Materials Science and Technology (INSTM), Via G. Giusti 9, 50121 Firenze, Italy; 6 Department of Physics and Astronomy and LENS, University of Florence, Via G. Sansone1, 50125 Sesto Fiorentino (FI), Italy; 7 Nuclear Safety, Security and Sustainability Division, Fusion and Technology for Nuclear Safety and Security Department, Italian National Agency for New Technologies, Energy and Sustainable Economic Development (ENEA), Via Martiri di Monte Sole 4, 40129 Bologna, Italy

**Keywords:** perovskite, magnetron-sputtering, thin-films, cesium lead halides, amplified spontaneous emission

## Abstract

Due to their high optical efficiency, low-cost fabrication and wide variety in composition and bandgap, halide perovskites are recognized nowadays as real contenders for the development of the next generation of optoelectronic devices, which, among others, often require high quality over large areas which is readily attainable by vacuum deposition. Here, we report the amplified spontaneous emission (ASE) properties of two CsPbBr_3_ films obtained by single-step RF-magnetron sputtering from a target containing precursors with variable compositions. Both the samples show ASE over a broad range of temperatures from 10 K up to 270 K. The ASE threshold results strongly temperature dependent, with the best performance occurring at about 50 K (down to 100 µJ/cm^2^), whereas at higher temperatures, there is evidence of thermally induced optical quenching. The observed temperature dependence is consistent with exciton detrapping up to about 50 K. At higher temperatures, progressive free exciton dissociation favors higher carrier mobility and increases trapping at defect states with consequent emission reduction and increased thresholds. The reported results open the way for effective large-area, high quality, organic solution-free deposited perovskite thin films for optoelectronic applications, with a remarkable capability to finely tune their physical properties.

## 1. Introduction

In the last decade, halide perovskites have attracted attention thanks to their peculiar optical properties, making them ideal candidates as active materials for a high number of technological applications. Low-cost fabrication, high bandgap tunability, easy tuning of the composition and promising emission properties, such as high emission quantum yield and optical gain, are appealing characteristics for applications in sensors [1,2], light emitting devices (LEDs, lasers) [3,4,5,6,7,8,9,10], optical memories [11] and solar cells [12,13,14]. The first halide perovskite systems were hybrid organic/inorganic, based on methylammonium lead trihalide (MAPbX_3_, with X = Cl, Br, I), but their organic nature leads to chemical instability under UV light, high temperature, humidity and other extreme conditions [15,16]. In recent years, fully inorganic halide perovskites have contributed to overcome most of the drawbacks of hybrid systems, whilst maintaining their main optoelectronic properties [17,18,19]. The full control of the chemical composition, surface quality and sample size (transversal and lateral), warranted by several deposition techniques, allowed for an improvement in the structural, morphological and physical properties compared to hybrid systems [19,20]. In such a context, conceiving and developing new strategies for the production of efficient large-area (several cm^2^) active layers with advanced properties in terms of technological control and optical performances is currently an open scientific challenge [21,22,23,24]. 

Several approaches have been proposed for the deposition of bulk all-inorganic halide perovskite active layers, among which, the solution-processing of precursors spin-coated on a substrate is the most common technique [25,26,27,28,29]. This is an easy, relatively fast, room temperature process, not requiring specific deposition conditions such as vacuum. It is, therefore, a low-cost and time-saving method. However, there are huge restrictions due to the spin-coating process, such as high material consumption, low control of the resulting film purity and the fact that they cover a limited area [21,22,30]. The toxicities of the employed organic solvents are an additional, nontrivial issue. 

Particular attention is currently being paid to CsPbBr_3_ thin films, which combine the improved stability of fully inorganic perovskites with efficient green emission, and are particularly interesting for applications in light emitting devices, such as LEDs and lasers. However, while solution processed CsPbBr_3_ nanocrystals films show efficient amplified spontaneous emission (ASE) at room temperature [31,32], bulk polycrystalline CsPbBr_3_ thin films deposited from solution show ASE only up to about 150 K [25], while ASE at room temperature is observed only after post-deposition recrystallization [29].

These results thus have stimulated the development of dry deposition techniques, based on vapor deposition under vacuum conditions, aiming to deposit large-area and high-purity samples showing ASE at room temperature. Thermal evaporation and pulsed laser deposition have been recently demonstrated to allow the deposition of high-quality films. CsPbBr_3_ films obtained by vacuum-based co-deposition of CsBr and PbBr_2_ precursors exhibited an ASE threshold down to 35 μJ/cm^2^ at room temperature under nanosecond pumping [33]. Furthermore, CsPbBr_3_ thin films fabricated by using a single-step physical technique, pulsed laser deposition (PLD), demonstrated stimulated emission with a low pump threshold at room temperature (18 μJ/cm^2^) under femtosecond pumping [34]. These results thus show the improved ASE properties of films deposited by dry techniques with respect to solution processed films.

Nevertheless, some limitations remain, such as the need for thermal sources, often double making the stoichiometry modification difficult, thus hindering the potential tuning of the sample properties. In this concern, antisolvent vapor crystallization (AVC) techniques, based on ion exchange supported by annealing processes, have opened the way for a further good control of the film thickness and composition, showing emission but not ASE [35].

In the last few years, the realization of high-quality thin films by single-step RF-magnetron sputtering promises to overcome the current limitations of thermal evaporation. It consists of a single step process, where a target, previously prepared by mixing and pressing an adjusted molar ratio of precursor milled salts, is sputtered on a substrate at room temperature. This technique allows to obtain thin films with minor roughness and better uniformity, compared to the ones obtained by normal solution methods [15]. Desired film thickness is reached by using a quartz microbalance located in the sputtering chamber, and the resulting stoichiometry is easily adjusted by tuning the molar ratio of the starting precursors salts [21,22,23]. The optical properties of sputtered films have been studied in terms of steady-state and time-resolved emission up to room temperature. They show excitonic emission and slight exciton localization (a few meV) dynamics at low temperature (10 K), revealed by decay times on the order of tens of picoseconds [22]. To date, the investigation of emission properties of fully inorganic perovskite films deposited by RF-magnetron sputtering has been limited to spontaneous emission, while the stimulated emission properties, fundamental information for its possible applications in lasers, are still unknown.

In this work, we demonstrate for the first time the amplified spontaneous emission from two CsPbBr_3_ films obtained by single-step RF-magnetron sputtering, deposited from different target compositions. The films show ASE in a broad range of temperatures (up to 270 K) with thresholds down to about 100 µJ/cm^2^ under nanosecond pumping. We demonstrate that the temperature dependence of the ASE threshold is due to the thermal activation of exciton detrapping up to T = 50 K, leading to a threshold decrease between 10 K and 50 K, followed by the thermal activation of non-radiative processes, progressively increasing the threshold up to the ASE disappearance. We also show that the optical performance could be readily controlled by modulating the initial stoichiometry of the target, affecting both the ASE threshold value and the temperature range in which ASE is present. We explain these results on the basis of a better stoichiometry of the sputtered samples when an excess of CsBr is present in the target, which was closer to the expected stoichiometry and confirmed by the XPS analysis, probably decreasing the defects state density. The reported results highlight the applicability of RF-magnetron sputtering in the deposition of CsPbBr_3_ uniform films able to support optical gain and stimulated emission. Furthermore, the versatility of the method allows for a fine tuning of the optical properties by appropriate adjustments of the target composition. This can open the way for the fabrication of large-area active layers in the future, for optoelectronic applications such as sensors, LEDs and solar cells.

## 2. Materials and Methods

### 2.1. Thin Film Fabrication

CsPbBr_3_ films were deposited using a Korvus HEX system (Korvus Technology Ltd., Newington, UK). The sputtering process was performed with argon plasma at a rate of 0.05 nm/s for up to 500 nm thick deposits, and monitored using a quartz cell microbalance located in the deposition chamber. To guarantee a homogeneous deposition thickness, the substrates were fixed on a rotating sample holder set to 10 rpm. S1 and S2 were obtained under the same sputtering conditions (RF power 20 W and an argon flow of 35 sccm). Each sample was deposited on two different substrates: a soda-lime glass slide and a silicon wafer. The samples on silicon were used for the SEM images.

### 2.2. Morphological and Structural Characterization: SEM, and XRD

Scanning electron microscopy (SEM) images were collected using a high-vacuum tungsten filament microscope (JEOL Ltd., Tokyo, Japan, model JSM-6480LV, dedicated software: SEM/JSM 5000) with a working bias of 20 kV.

The crystallinity of the samples was determined by X-ray diffraction (XRD) analysis performed by means of a diffractometer (Bruker Corporation, Billerica, MA, USA, model D8) working in Bragg–Brentano mode.

### 2.3. Chemical Characterization: XPS

The XPS instrument was a VSW Scientific Instrument Limited model TA10 (Manchester, UK) equipped with a non-monochromatic X-ray source, working in this case with an Al cathode, 144 W power and a VSW Scientific Instrument Limited model HA100 (Manchester, UK) hemispherical analyzer with a 16-channel detector. For each sample, a survey scan was acquired to select the range in which the high resolution scans were acquired.

### 2.4. Absorption and ASE Characterization

The UV−Vis absorption spectra were acquired using a spectrophotometer (PerkinElmer Inc., Waltham, MA, USA, model UV−Vis Lambda 900) equipped with an integrating sphere.

Photoluminescence and amplified stimulated emission (ASE) measurements were conducted by exciting the films with a nitrogen laser (Lasertechnik Berlin GmbH, Berlin, Germany, model MNL 100), delivering 3 ns pulses at 337 nm with a peak energy up to 155 µJ, and focused on a rectangular stripe (4 mm × 80 μm). The laser excitation density was changed by a variable neutral filter. The film emission was collected in waveguide configuration from the sample edge by means of an optical fiber coupled to a spectrometer (Teledyne Princeton Instruments, Trenton, NY, USA, model ACTON SpectraPro-750) equipped with an air-cooled silicon CCD (Andor Technology Ltd., Belfast, Northern Ireland, model iVac). The spectral resolution was about 0.5 nm.

Films were excited under vacuum (with a pressure of about 10^−2^ mbar) at different temperatures in the range from 10 to 300 K using a closed cycle He cryostat. 

The method employed to determine the ASE threshold was based on the excitation density dependence of the PL spectra. In particular, the ASE threshold was defined as the excitation density at which the spectral shape starts to change due to the appearance of an ASE band. This procedure, known as the visual method [36], seems to be the best one to provide the lowest threshold values due to the appearance of the early stage ASE emission in a straightforward way.

## 3. Results and Discussion

The investigated samples consisted of two CsPbBr_3_ films, obtained by RF-magnetron sputtering (fabrication procedure is reported in the Section 2), with a thickness of 500 nm (obtained by profilometer measurements). 

Sample 1 (hereafter called S1) was obtained by using a 1:1 mol/mol target of CsBr (purity 99%, Chempur Feinchemikalien und Forschungsbedarf GmbH, Karlsruhe, Germany) and PbBr_2_ (purity 98%, Fluorochem, Dublin, Ireland). After analysis of the sample, the presence of perovskite in the deposited film was confirmed but the stoichiometry showed a deficiency of cesium (see Table 1). To overcome this problem and obtain a thin film with a composition closer to the expected outcome, a second target was prepared with a composition of 1.15:1 mol/mol under the same deposition conditions, and this was Sample 2 (hereafter called S2).

**Figure 1:**
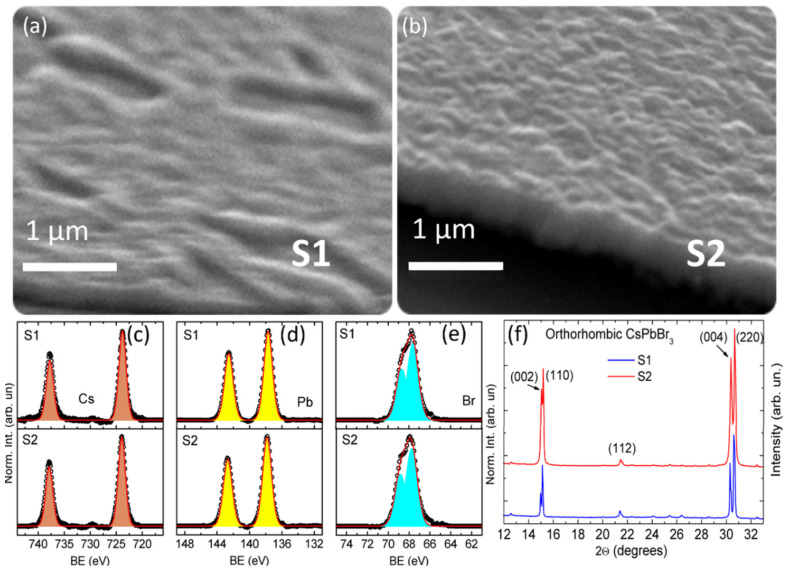
(**a**) SEM micrograph of a random area of sample S1. The formation of micro-sized superstructures and valleys is shown. (**b**) SEM micrograph of a random area of sample S2 (with corrected target composition), showing a flat and uniform film, differentiating S2 from S1. (**c**–**e**) XPS high-resolution spectra of S1 (top) and S2 (bottom), from left to right the regions of cesium, lead and bromide are reported. (**f**) XRD patterns of samples S1 and S2, showing good agreement with the orthorhombic phase of CsPbBr_3_ for both.

In order to verify the correct deposition of perovskite, SEM, XPS and XRD analyses of the two samples were performed (Figure 1). The deposits were composed of almost uniformly distributed crystalline aggregates. Even though the surfaces of the samples were free of voids and fully covered by perovskite (see XPS results, Figure 1c–e), sample S1 (Figure 1a) presents randomly distributed micrometer-sized superstructures and valleys, while sample S2 (Figure 1b) features a more uniform thickness distribution. The defective stoichiometry of sample S1 (cesium deficiency) could account for these morphological differences. Notably, in both samples, the XRD patterns (shown in Figure 1f) are consistent with the orthorhombic phase of CsPbBr_3_ at room temperature [37,38,39], whereas the XPS high-resolution spectra show that there are no differences between sample S1 and sample S2 in terms of chemical shift.

In Table 1 we report the elemental composition of the two samples obtained by fitting of the high-resolution XPS spectra. From the reported data, it is possible to notice the low amount of cesium in sample S1 with respect to the expected composition. By increasing the amount of CsBr in the target, the stoichiometry of the sputtered film (sample S2) approaches the expected one regarding the amount of Cs, whereas a decrease in Br is observed, ascribed to the lower relative Br content with respect to Cs in the used target. The residual presence of oxygen in the deposition chamber could also result in unwanted compounds of both cesium and lead, which present chemical shifts close to oxides and halides, thus are not readily observed in XPS spectra. The slight deviations in the final film composition with respect to the expected one (Table 1) can be attributed to different phenomena typically present in the sputtering processes occurring both on the target and the substrate surface, namely target erosion, sticking coefficient variations, re-sputtering, a small contribution in the transport phase due to the different mean free paths of the atoms involved [40], as well as the difference between angular and energy distributions of the sputtered flux for each target element [41].

The room temperature absorption spectra of the two samples (see Figure S1 in Supporting Information) show an absorption edge around 525 nm and a clear exciton resonance, peaked at about 513 nm and 516 nm for S1 and S2, respectively, consistent with the typical absorption range of CsPbBr_3_ films [21,25,34]. Despite the comparable thickness, it is interesting to observe that the absorbance of sample S2 is lower than that of sample S1 over the whole absorption spectral range (about 2.4 times), evidencing a lower density of absorbing sites, and thus also of emitting sites.

**Figure 2:**
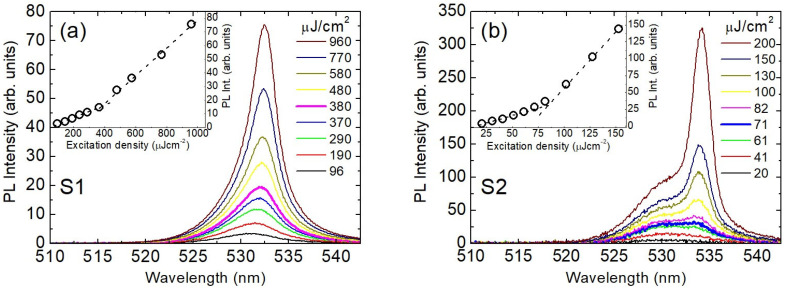
Excitation density dependence of the emission spectra of sample S1 (**a**) and S2 (**b**) recorded at 50 K, showing the progressive ASE band appearance. The thickest lines show the spectrum at the excitation density closest to the ASE threshold. Insets: excitation density dependence of the emission intensity at the peak ASE wavelength (symbols). Dotted lines are a guide for the eyes.

**Figure 3:**
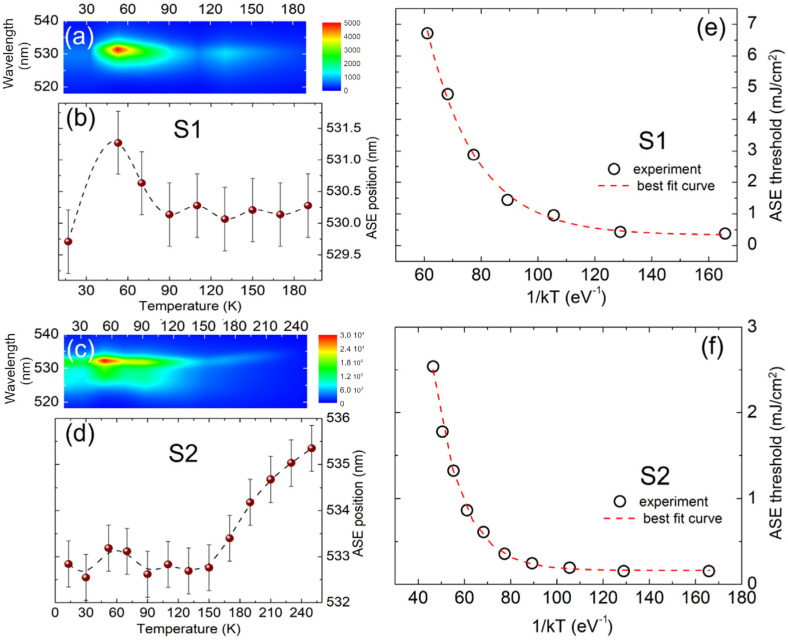
(**a**,**c**) 2D map of the PL spectra above the temperature for sample S1 (**a**) and S2 (**c**) recorded by exciting the films at an excitation density corresponding to the double of the threshold at each temperature. (**b**,**d**) Evolution of the ASE peak position (symbols) as a function of the temperature. Error bars are defined by the spectral resolution of the spectrometer used in the experiments (0.5 nm). The dotted lines are a guide for the eyes. (**e**,**f**) Arrhenius plots of the extracted ASE thresholds in sample S1 (**e**) and S2 (**f**) (symbols) and corresponding best fit curves (dotted lines) to Equation (1).

The ASE properties of the samples were investigated by measuring the excitation density dependence of the emission spectra at different temperatures between 10 K and room temperature. Typical results are reported in Figure 2, where the spectra collected at 50 K are shown. They show evidence of a clear line-shape variation and spectral narrowing upon an increase in the excitation density, typical of amplified spontaneous emission (see also Figure S2). The ASE band is peaked at about 531.5 nm in sample S1 and 533 nm in sample S2 (see Figure 3b,d for the temperature dependence), both falling on the red side of the spontaneous PL (peak at around 530 nm), where the self-absorption of the emitter species is smaller than that measured at the peak wavelength of spontaneous emission. This hypothesis is rationalized by comparing the absorbance values at the absorption peak (α_abs_) with those estimated at the ASE peak, α_ASE_, considering a spectral resolution of 0.5 nm (see Figure 3). We define the percent ratio, R = 100 (α_ASE_/α_abs_), in both the samples, for which we expect similar values, as in this case self-absorption is the main process defining the spectral window of ASE. We find that R1 = 21.9 ± 1.2 and R2 = 19.7 ± 1.4 (for sample S1 and S2, respectively), showing very good agreement between them, confirming that the ASE position is strictly related to the minor self-absorption contribution. The ASE appearance in the spectra leads to a clear increase in the slope of the emission intensity dependence on the excitation density (see insets of Figure 2) in correspondence with the ASE threshold excitation density (D_th_), which was about 380 µJ/cm^2^ and about 70 µJ/cm^2^ for sample S1 and S2, respectively.

The effect of the temperature on the ASE of the samples was investigated by determining the ASE threshold and the emission spectra at an excitation density of 2*D*_*th*_. The spectrum of sample S1 (see Figure 3a) show a strong ASE intensity increase between 10 K and 50 K while, as the temperature is further increased, a progressive decrease in intensity is observed up 190 K, where finally the ASE disappears for higher temperatures. Similar behavior is visible in the spectra of sample S2 (see Figure 3c), with an increase in intensity up to 50 K followed by a progressive decrease at higher temperatures up to the ASE loss above 270 K. Remarkably, sample S2 shows ASE in a much wider temperature range, with a maximum temperature at which ASE is visible very close to room temperature.

Concerning the temperature dependence of the ASE threshold, in sample S1 we observe (see Figure S3a) a clear initial decrease between 10 K and 50 K, followed by a weak increase up to about 90 K, and then a progressive increase at higher temperatures up to the loss of ASE. Sample S2 shows instead (see Figure S3b) a quantitatively smaller decrease of the threshold value up to 50–70 K, followed by an almost constant value up to 130 K and a final progressive increase up to 270 K.

A comparison between the samples shows that the ASE threshold of S2 is always much lower than S1 at all the investigated temperatures, with an S2/S1 ratio ranging from about 2.4 (70–90 K) to about 16 (10–50 K). At low temperature, the observed dynamics are consistent with a detrapping process from defect states (as further discussed later). In order to study the thermal processes affecting the ASE threshold above 50 K (see Figure 3e,f), we analyzed the data by applying the following model. We rationalized the stimulated emission process by considering a 3-level scheme (see Figure S4), a well-established scenario for perovskites [42], from which we define the temperature dependence of the ASE thresholds:

(1) $$D_{th}=D_{0}+D_{1}e^{-\frac{E_{a}}{kT}}$$

where *E_a_* is the activation energy, *k* is the Boltzmann constant and *D_th_*, *D*_0_ and *D*_1_ result from the complete calculus reported in the Supporting Information. The best fit curves of the experimental data to Equation (1) excellently reproduce the data (see Figure 3e,f) to give a best fit activation energy of 57 ± 4 meV and 81 ± 3 meV for S1 and S2, respectively.

**Figure 4:**
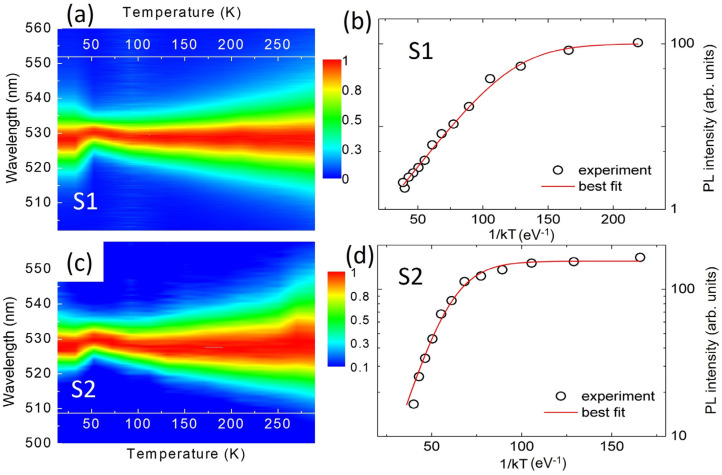
(**a**,**c**): 2D maps of the normalized emission spectra obtained at an excitation density one-half below the threshold. The detrapping process up to 50 K and the intrinsic nature of the emission at higher temperatures is evident. Temperature-induced broadening is also evident. (**b**,**d**) Arrhenius plot of the PL intensities of sample S1 (**b**) and S2 (**d**) as a function of the temperature (symbols), analyzed for T > 50 K. Continuous lines are the best fit curves to Equation (2).

Previous studies carried out on similar systems by time-resolved experiments have highlighted the occurrence of a thermally induced variation in the population of the starting level in the radiative relaxation transition [22]. With the aim of studying this hypothesis in our samples we have also analyzed the temperature dependence of the non-amplified spontaneous emission intensity (the 2D map of the temperature evolution of the normalized spectra is shown in Figure 4a,c). In this case, we measured the emission spectra at an excitation density of one-half of the threshold (*D_th_*/2) at each temperature, then scaling the intensity proportionally to the excitation density (assuming a linear dependence of the emission intensity on the excitation density at low excitation levels) (see Figure 4b,d). The temperature dependence of the spectrally integrated emission intensity (see also Figure S5) clearly shows a close similarity with the ASE threshold thermal behavior. First, for sample S1, we observe a clear intensity increase (about 2-fold) between 10 K and 50 K, corresponding to the temperature range of initial ASE threshold decrease. At higher temperatures, an initial weak PL quenching is observed, followed by a stronger quenching up to room temperature. A similar correlation is also observed for sample S2, showing a weakly temperature-dependent emission intensity up to about 110 K, followed by a thermally activated quenching up to room temperature, in the range in which the ASE threshold increase is present. Figure 4b,d shows the experimental data as a function of 1/kT (for T > 70 K, the whole dataset is shown in the Supporting Information, Figure S3). Although in both samples heavy quenching up to room temperature is present, sample S2 shows higher emission intensity and a smaller signal decrease with respect to sample S1 (about one order of magnitude above the whole temperature range), indicating a higher final quality of the film in terms of crystal purity when an excess of CsBr is applied to the starting sputtered target.

A quantitative analysis of the previous features has been performed by a best fit of the emission intensity temperature dependence to the following Equation (2). It can be easily obtained from the temperature dependence of population of the starting level of the radiative transition, as described before:

(2) $$I\left(T\right)=\frac{I_{0}}{1+\frac{\tau_{0}}{\tau_{T}}e^{\frac{-E_{a}}{kT}}}$$

where *I*_0_ is the ideal emission intensity at 0 K, $\tau_{0}$ is the temperature-independent part of the excited state lifetime and $1/\tau_{T}$ is the temperature-dependent nonradiative rate. The experimental data are well reproduced (see Figure 4b,d) for a best fit value of the activation energy of 46 ± 1 meV and 90 ± 9 meV for S1 and S2, respectively. These values are close to the corresponding activation energies for the threshold increase at high temperature (Figure 3). This basically indicates that the thermally activated process affecting the threshold and the emission intensity is the same.

Concerning the origin of the observed temperature dependence, we must separate the discussion to two temperature ranges: a low temperature range of up to 50 K, and a second temperature range for T > 50 K. Both the samples show similar behavior up to 50 K, consisting of a progressive red-shift of the emission, together with a shrinking of the spectra below threshold (see Figure S6), a decrease in the ASE threshold (Figure 3e,f) and a PL intensity increase (more evident for sample S1). Above 50 K, we observe a slight blue-shift, an increase in the broadening and a decrease in the intensity, in line with e–h recombination from intrinsic states [41,42,43].

Concerning the low temperature range, we observe that the broad and weak emission is often reported in lead halide perovskites, and it is the typical signature of emission from carriers localized in trap states [43,44,45]. With an increase in temperature, thermally activated carrier detrapping promotes the formation of a free-exciton, which results in a progressive red-shift of the emission (see scheme of Figure S4), a narrowing in emission and an increase in intensity. The detrapping temperature range mainly depends on the localization energy at 10 K. In our case, we observe detrapping effects up to about 50 K, allowing estimation of a localization energy on the order of 4 meV. Above 50 K, the thermal behavior of the emission intensity (see Figure 4) is affected by non-radiative processes characterized by very different activation energies between the two films studied, also reflected by a significant consistency in the thermal behavior of the ASE thresholds and their activation energies for T > 50 K (Figure 3b,d). In this regard, studies where thermally induced quenching of the emission and/or the ASE threshold are observed [25] have argued that the main contribution to the quenching could come from exciton dissociation induced when thermal energy approaches the binding energy of exciton. This hypothesis, however, should imply similar activation energies in both our samples due to their similar flat morphology and chemical composition. Alternatively, other studies have shown that PL thermal-induced quenching (with activation energies similar to those reported in this work) was due to carrier migration among defect states, assisted by thermally induced higher carrier mobility [46]. In such a context, the different nature and density of the defects between the samples could determine variations in the observed activation energies and the degree of signal quenching over the whole analyzed temperature range, with consequent effects on the ASE performance at high temperature. The activation energy in sample S1 is lower than sample S2, so quenching is evident at lower temperatures, leading to an ASE threshold increase up to the ASE loss at temperatures lower than those of sample S2. Moreover, it is worth noting that the degree of signal quenching (defined as the signal ratio between the maximum and minimum intensity recorded) in S2 is about 15.5, whereas in S1 it is 41.5. This indicates that sample S2 seems to be of better quality, probably induced by the excess CsBr applied in the starting target, leading to a minor signal quenching over the temperature range and a higher activation energy.

Remarkably, the emission energy of both the films when excited above the ASE threshold, reported in Figure 3b,d, follows the same temperature dependence of the PL below the threshold in terms of peak position (see Figure S6). They show an initial red-shift up to 50 K, followed by a rapid blue-shift and a constant trend in the temperature range 70–150 K. Sample S2 (showing ASE up to higher T) undergoes a further red-shift above 150 K (up to ASE loss at about 270 K). The origin of this shift is a topic of debate and can be ascribed to the exciton–exciton scattering occurring at high excitation levels, a the well-recognized cause of ASE in perovskite materials similar to ours [34].

Overall, we can conclude that the better ASE properties of sample S2 come from a combination of different features, i.e., a better morphology uniformity, a lower self-absorption and a lower trapping contribution to the emission quenching. Our results show that lead halide deposition by RF-sputtering has wide possibilities in engineering due to the films emission properties, which can be altered by simply acting on the precursors relative content in the target. From this point of view, one step RF-magnetron sputtering allows for the effective control of the desired film composition and overcomes the limit of other vacuum-based deposition techniques, such as thermal evaporation, which often require double crucibles in order to control the deposition stoichiometry.

## 4. Conclusions

In conclusion, we have demonstrated for the first time amplified spontaneous emission in CsPbBr_3_ thin films obtained by single-step RF-magnetron sputtering. Optical gain is observed up to almost room temperature (with a threshold down to 100 µJ/cm^2^ around 50 K) and the results are significantly sensitive to the composition of the target (an up to four times improvement in the ASE performance is observed when an excess of CsBr is used). Our results open the way for the next generation of gain media with easily controllable optical features by exploiting the versatility of RF magnetron sputtering. This technique, which is relatively novel for obtaining perovskite thin films, allows for the fabrication of high quality, large-area films with tunable optical properties and could be an important core technique for the development of next generation of electro-optical devices.

## Figures and Tables

**Table 1:** Elemental composition of S1 and S2 samples determined from the XPS spectra, and the expected composition of a CsPbBr_3_ sample.

Element	Cs	Pb	Br
S1 composition ^1^	15%	29%	56%
S2 composition ^1^	23%	30%	47%
Expected composition ^1^	20%	20%	60%

^1^ atomic percent.

## Data Availability

The data presented in this study are available on request from the corresponding author.

## Supplementary Materials

The following supporting information can be downloaded at: https://www.mdpi.com/article/10.3390/nano13020306/s1, Figure S1: absorption spectra; Figure S2: PL spectra variation due to ASE; Figure S3: complete ASE threshold temperature dependence; Figure S4: energy levels scheme; Figure S5: complete PL intensity temperature dependence; Figure S6: PL peak wavelength and FWHM temperature dependence. Model of the ASE threshold temperature dependence.
