# Supplementary material for: Temperature-Dependent Amplified Spontaneous Emission in CsPbBr3 Thin Films Deposited by Single-Step RF-Magnetron Sputtering
Source: Nanomaterials (Basel). 2023 Jan 11;13(2):306. doi: 10.3390/nano13020306 (PMC9866928; doi:10.3390/nano13020306)
Supplement: Supplementary file 1 [file nanomaterials-13-00306-s001.zip › nanomaterials-2146348-supplementary.docx]

Supporting Information

# Temperature dependent amplified spontaneous emission in CsPbBr3 thin films deposited by single-step RF-magnetron sputtering

*Giovanni Morello, Stefania Milanese, Maria Luisa De Giorgi, Nicola Calisi, Stefano Caporali, Francesco Biccari, Naomi Falsini, Anna Vinattieri and Marco Anni*

**Figure S1**: Absorption spectra of the two samples studied recorded at room temperature.

**
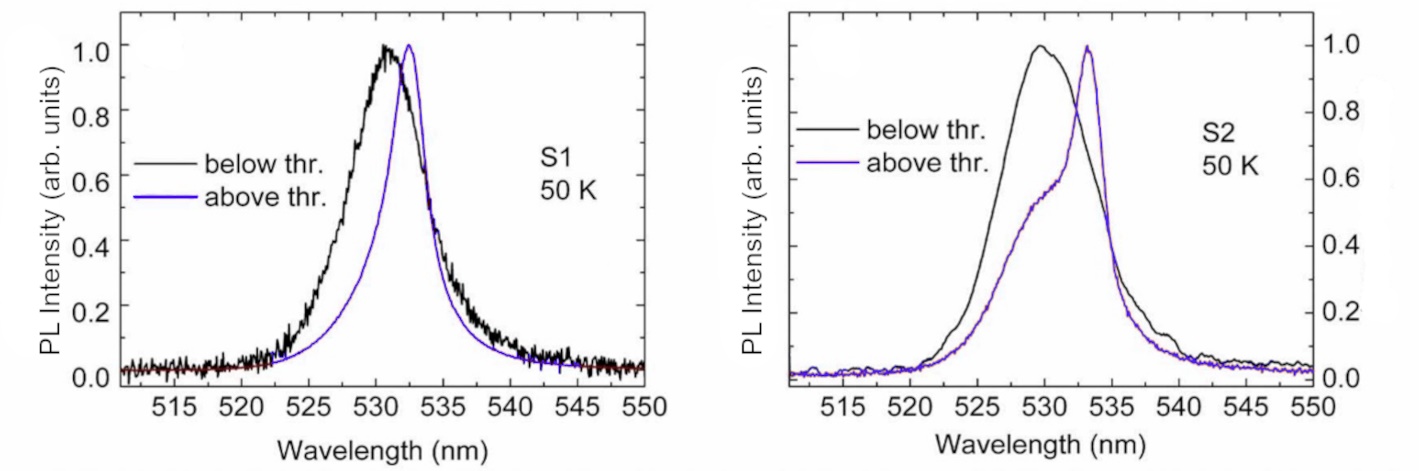
 Figure S2**: Comparison of the normalized emission spectra obtained at an excitation below and above threshold for both the samples studied, recorded at 50 K.

**
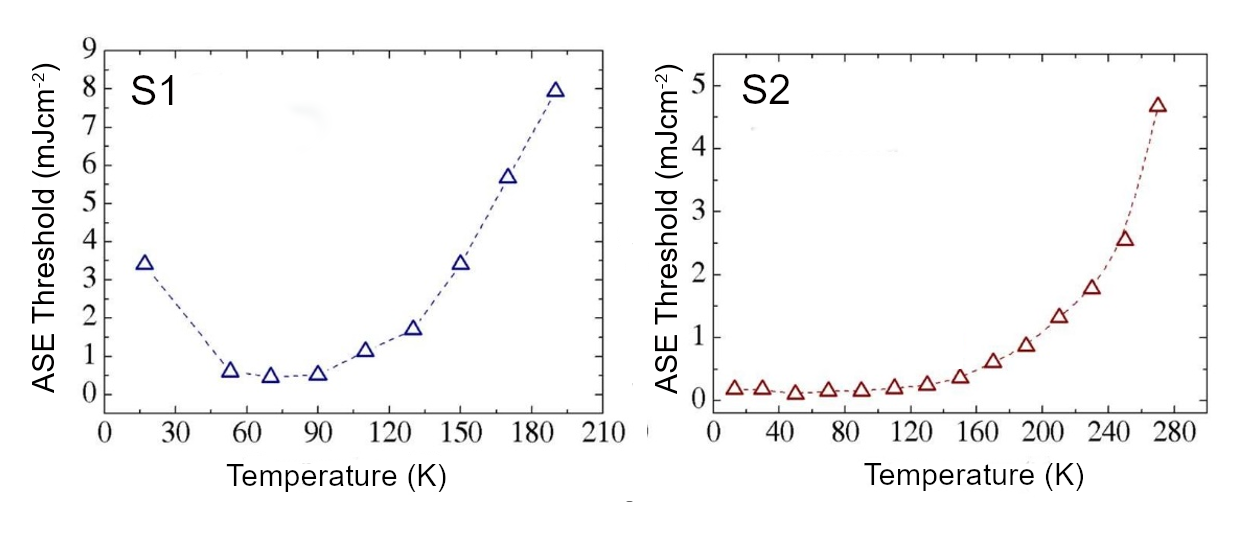
Figure S3**: Complete dataset of the extracted thresholds in the samples studied, as a function of the temperature.


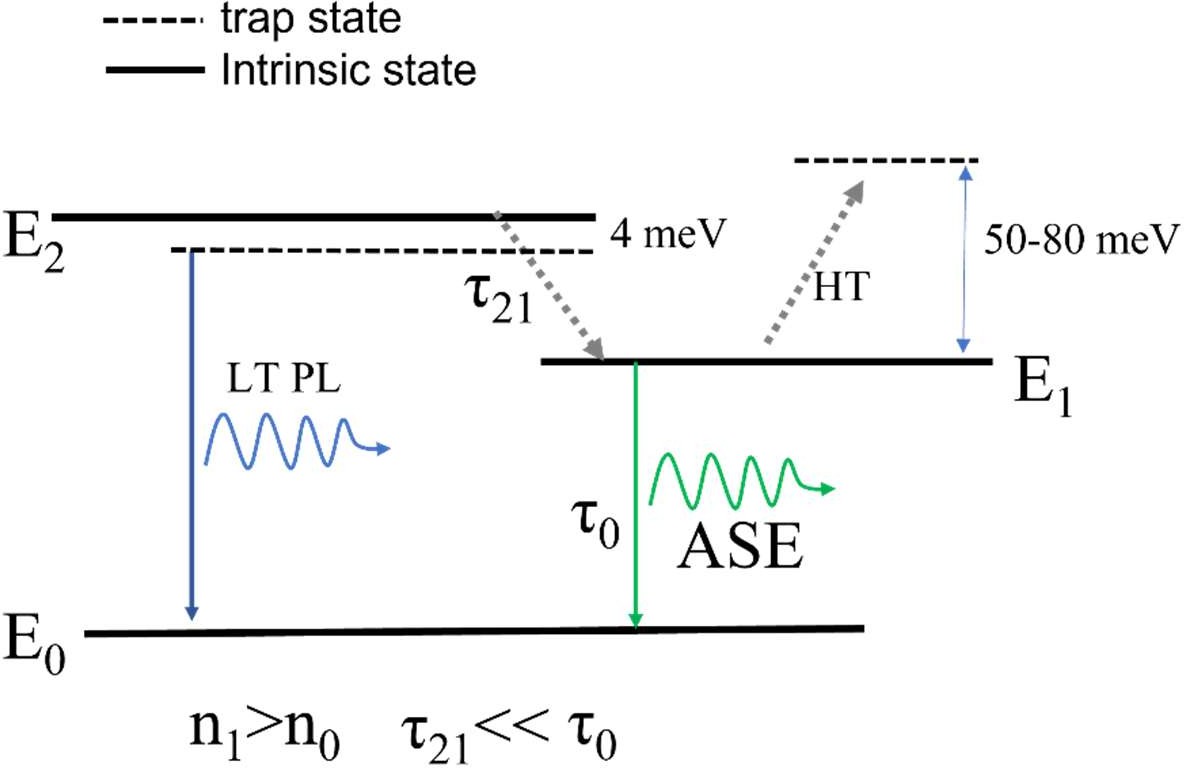


**Figure S4**: 3-level scheme, showing the main processes occurring during ASE action at different temperature ranges.


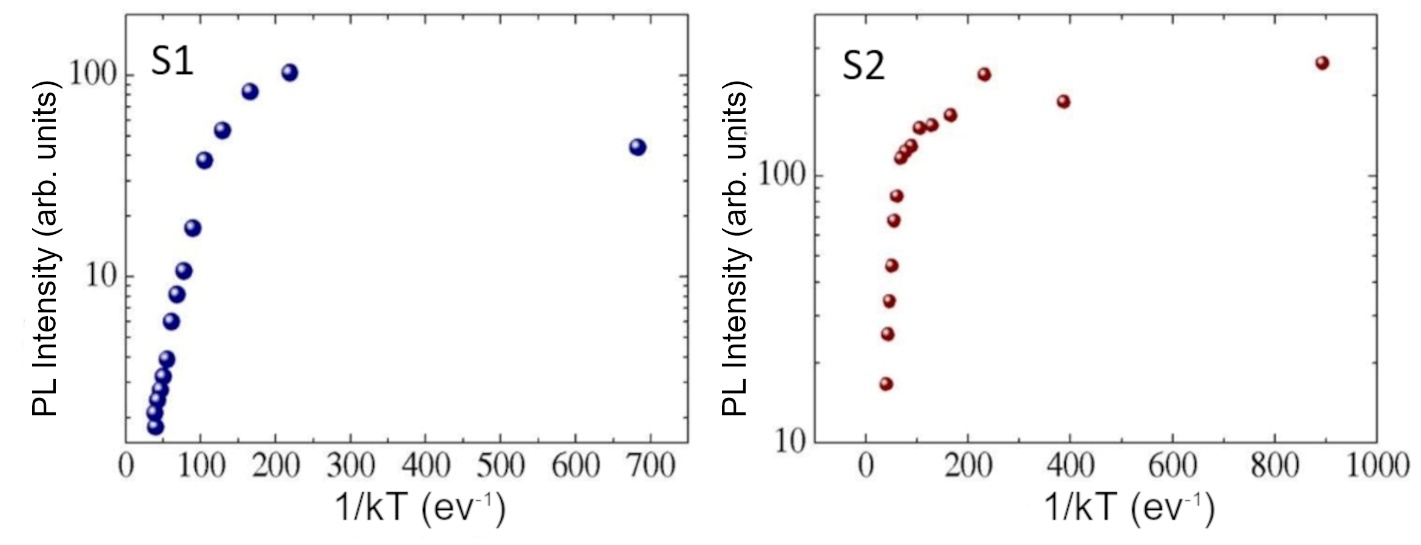


**Figure S5**: complete dataset of the PL intensities vs. temperature.


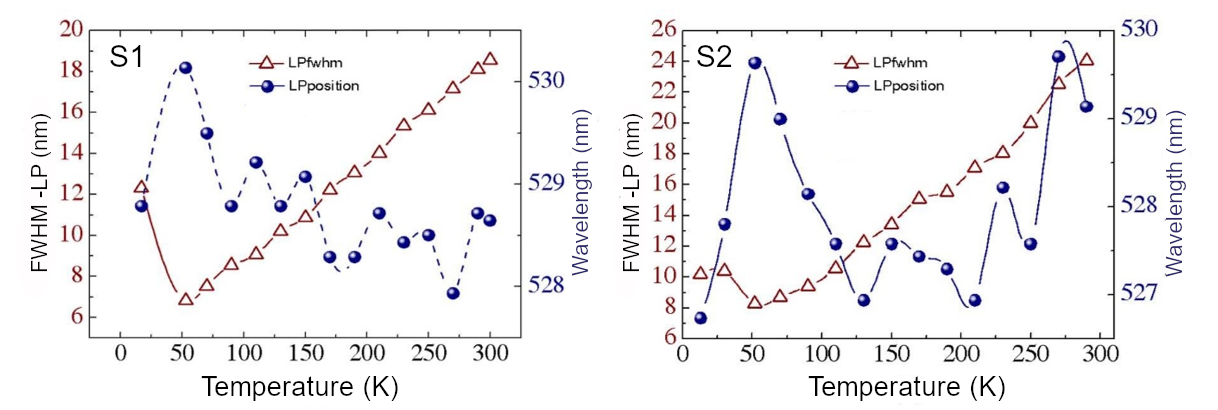


**Figure S6:** PL position and FWHM as a function of the temperature in both the samples S1 and S2, when excited at one half of the threshold density.

# Model of temperature dependence of ASE Thresholds in a 3-level system

If N is the whole population (volume density, cm^-3^) and n0 and n1 are the populations of the lower and upper states, respectively (with N = n0+n1), the condition for population inversion is reached when a half of the carriers (N/2) occupies the upper state, i.e. n1=N/2.

The amplification process starts when

$\sigma\left( {2n}_{1}-N \right)>\alpha\Leftrightarrow{2n}_{1}-N=\frac{\alpha}{\sigma}$ (1)

where α represents the losses coefficient and σ is the gain cross-section. Let n1th be the population level at the threshold condition; the consequent depopulation rate at the threshold pumping can be expressed as

$\frac{n_{1th}}{\tau}=\frac{1}{2}\left( \frac{\alpha}{\sigma}+N \right)\frac{1}{\tau}$ (2)

Here, $\frac{1}{\tau}=\frac{1}{\tau_{0}}+\frac{1}{\tau_{nr}}$ is the total decay rate at the threshold condition, $\tau_{0}$ is the intrinsic transition lifetime and $\tau_{nr}$ is the lifetime of a non-radiative process, a parameter accounting for eventual thermally activated processes, such as thermally induced carrier trapping/detrapping, exciton thermal dissociation, exciton-exciton scattering, carrier thermal escape from the material, all of them detrimental for efficient ASE .^[32]^ In fact, for a defined thermally induced non-radiative process (characterized by the lifetime $\tau_{T}$ and an activation energy $E_{a}$), the rate 1/τ_nr_ is expressed as

$\frac{1}{\tau_{nr}}= \frac{1}{\tau_{T}}e^{-\frac{E_{a}}{kT}}$ (3)

Where k is the Boltzmann constant.

In steady state regime (our pump pulse is much longer than the ASE lifetime) the excited state depopulation rate $\frac{n_{1th}}{\tau}$ is equal to the excitation rate g_0th_.

As the pump rate is directly proportional to the excitation density we finally have D_th_=Cg_0th_ and, substituting Equation 3 in Equation 2 and compacting the temperature independent terms in $D_{0}=C\frac{1}{2}\left( \frac{\alpha}{\sigma}+N \right)\frac{1}{\tau_{0}}$, $D_{1}=C\frac{1}{2}\left( \frac{\alpha}{\sigma}+N \right)\frac{1}{\tau_{T}}$, and $D_{th}=C\frac{n_{2th}}{\tau}$:

$$D_{th}=D_{0}+D_{1}e^{-\frac{E_{a}}{KT}}$$
